# Challenges for esketamine nasal spray in China: use and management

**DOI:** 10.3389/fphar.2024.1429435

**Published:** 2024-10-01

**Authors:** Jianhong Wu, Jun Gu, Linghe Qiu, Xin Jin, Zhenhe Zhou

**Affiliations:** ^1^ The Affiliated Mental Health Center of Jiangnan University, Wuxi Central Rehabilitation Hospital, Wuxi, Jiangsu, China; ^2^ Northern Jiangsu People’s Hospital Affiliated to Yangzhou University, Yangzhou, Jiangsu, China

**Keywords:** challenges, esketamine nasal spray, China, use, management

## Introduction

**FIGURE 1:**
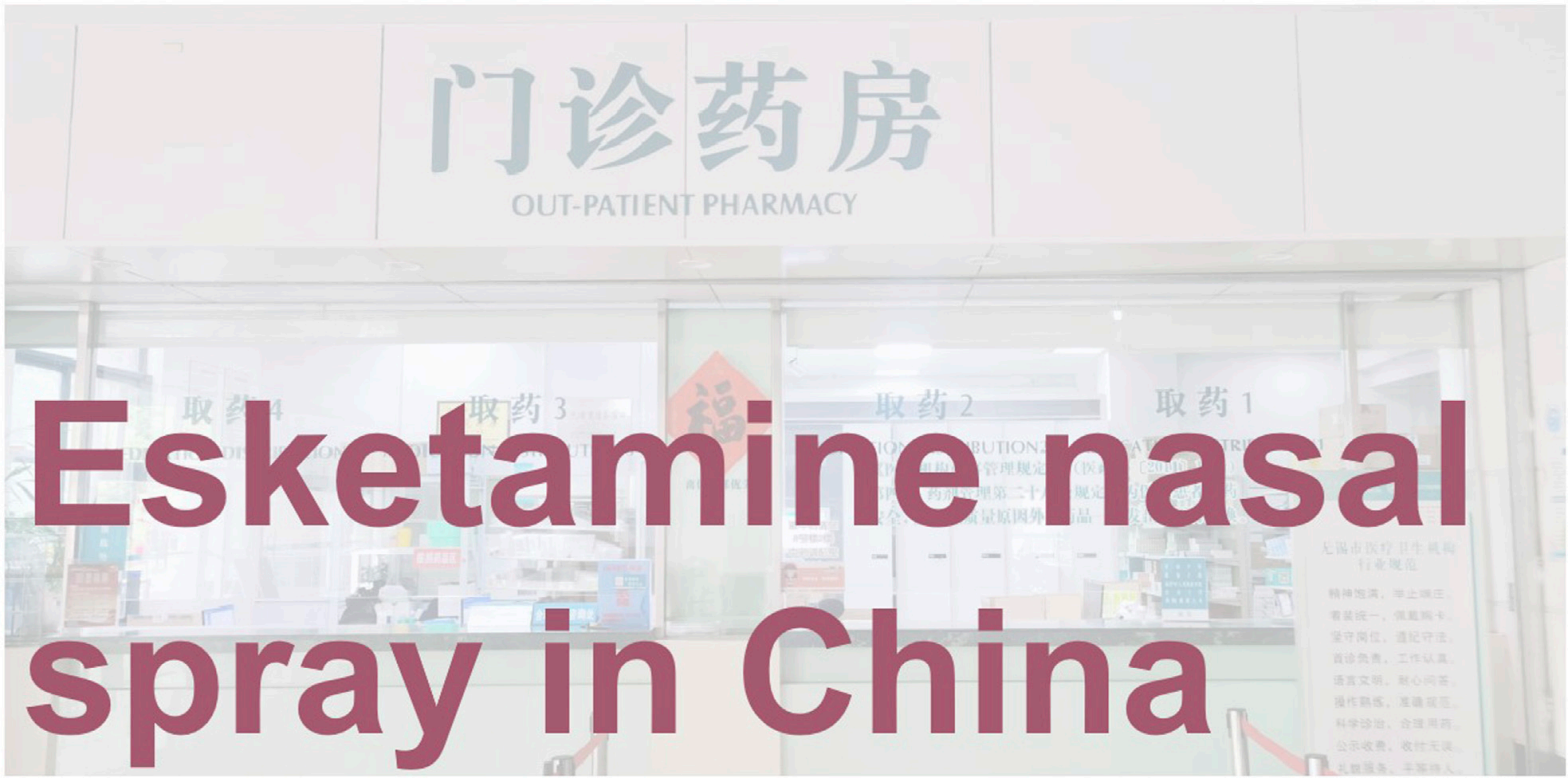
Esketamine nasal spray in China.

Depression is a common mental disorder characterized by persistent significant low mood and anhedonia, accompanied by different degrees of cognitive and behavioral changes. It is also one of the primary causes of disability and is considered the second leading cause of life disability in China (Lu et al., 2021). In March 2019, esketamine nasal spray was officially approved for sale in the United States, which means in the past 30 years, it is the first antidepressant based on a new mechanism (Su et al., 2023) designed to address the increasing prevalence of depression (Ross and Soeteman, 2020). After marketing in the United States for 4 years (Food and Drug Administration, 2019), esketamine nasal spray was finally approved in mainland China on 20 April 2023, and will gradually be promoted in various provinces and cities (Figure 1).

However, it can be expected that the administrative management of esketamine nasal spray in China will be extremely strict, particularly for clinical applications. China implements a classification management system for psychotropic drugs and divides them into Category I and II psychotropic substances according to drug dependence. According to the national regulatory rules, Category I psychotropic substances cause more dependence than Category II substances. Ketamine, known as “K powder” in China and named “Special K” as a party drug in the United States, is medically classified as a Category I psychotropic substance due to its potential addiction and abuse issues (Morgan et al., 2012). As it is an S-enantiomer of ketamine, esketamine nasal spray should also adhere to the guidelines followed for the management of Category I psychotropic substances, including its import, production, operation, and use, as confirmed in the drug management notification proposed by the National Medical Products Administration (NMPA) in May 2023. Due to its potential addiction, the use of esketamine nasal spray is subjected to the supervision of professional medical staff (Canuso et al., 2018). In the United States, the Food and Drug Administration (FDA) stipulates that esketamine nasal spray must be administered in medical settings under necessary monitoring conditions for specific registered patients (Reardon 2019). After medication administration, patients should be observed in the medical setting for 2 h before leaving, and they cannot take the nasal spray out of the clinic (Ross and Soeteman, 2020). Likewise, China’s management of this drug will be stringent. Similar to the control on other Category I psychotropic substances, medical institutions even need to recover the used nasal spray device simultaneously (Sun et al., 2023). This creates a contradictory “vicious cycle”: if regulations are strict, the use of esketamine nasal spray is restricted; if regulations are lax, there is a risk of addiction to esketamine nasal spray. The usage restriction and abuse liability will greatly limit the accessibility of esketamine nasal spray.

In addition to drug abuse risk, another reason for stringent management is its prominent adverse drug events (ADE) (Gastaldon et al., 2021). The FDA adverse event reporting system (FAERS) shows that specific adverse reactions, including dissociation, sedation, nausea, vertigo, anxiety, sensory delay, increased blood pressure, somnolence, headache, vomiting, feeling of intoxication, and suicidal ideation, are likely to occur following esketamine use (McIntyre et al., 2021; Guo et al., 2022). Among them, dissociation and sedation are the most common treatment-emergent adverse events (TEAE) associated with esketamine, along with perceptual disturbances, abnormal sensations, derealization, and depersonalization. Moreover, some new and clinically valuable potential ADEs such as flashback, tachyphylaxis, and autoscopy, have also been reported (Jiang et al., 2023). Flashback and autoscopy might be associated with the pharmacological mechanism of esketamine (Bowirrat et al., 2022), while tachyphylaxis may affect long-term effects. Tachyphylaxis refers to the phenomenon of diminishing efficacy, especially when administered continuously or repeatedly, which is a huge challenge for patients with long-term use (Sharma, 2019). In addition, cognitive dysfunction and impaired working memory under long-term use of esketamine should also be given special attention. Participants aged ≥65 years showed slowing of simple and choice reaction times during the optimization/maintenance period in the SUSTAIN III trial (Zaki et al., 2023), and further research is warranted to determine whether the observed slowing of reaction time possesses clinical relevance.

An analysis about the FAERS database shows that from 2016 to 2023, esketamine-related ADE reports increased annually, including hospitalization or extended hospital stay and death-related reports (Jiang et al., 2023). Special experiences such as euphoric mood, feeling of relaxation, and feeling drunk should be paid close attention to, which partly explains the risk of esketamine abuse and addiction: By antagonizing NMDA receptors, esketamine influences the balance of neurotransmitters such as 5-HT and dopamine, which plays a role in pleasure and reward system (Yagishita, 2020). Furthermore, although esketamine is used to alleviate suicidal behavior in patients with depression, the adverse risk of suicidal ideation has been reported (McIntyre et al., 2021). This may be partly attributed to mood swings (Nguyen et al., 2023) or abnormal fluctuations in neurotransmitters (Arazi et al., 2022) caused by esketamine. The resulting long-term impact and risks need to be further studied. Due to the particularity of nasal spray administration, problems such as nasal discomfort and drug monitoring procedure incorrectly performed arise (Jiang et al., 2023). Overall, medical staff should be familiar with common safety concerns, pay attention to and actively report long-term reactions and risks, and pragmatic prevention and management strategies are needed to reduce the patient burden and improve the acceptability of intranasal esketamine treatments.

## China’s prescription practice dilemma

The patient’s experience of adverse reactions, especially dissociation, sedation, and euphoric mood, could increase the possibility of drug abuse. Conversely, drug abuse could increase the incidence of adverse reactions (Baudot et al., 2022). The approach in the United States to reduce risks is following the risk evaluation and mitigation strategy (REMS), which is a set of risk management concepts. The United States is one of the first countries to introduce risk management in the pharmaceutical field. It has formed a relatively complete drug-risk management system from new drug application to drug use, including a medication guide, a communication plan, elements to ensure safe use, and an implementation system to help guide the prescribers, pharmacists, and patients (Nicholson et al., 2012). The FDA states that esketamine nasal spray should only be distributed to REMS-certified clinics and hospitals.

However, China does not have a comprehensive REMS network. Moreover, based on the strict crackdown on drug consumption and abuse, China will impose stringent restrictions on the use of esketamine nasal spray, which will greatly reduce its prescription (Liu et al., 2015; Chen, 2015). For instance, as stipulated in the Drug Administration Law, the Regulations on the Administration of Narcotic Drugs and Psychotropic Substances, and other laws and regulations, retailing of Category I psychotropic substances is not allowed, and they can only be issued at authorized medical institutions (Wen, 2012). The duration of each prescription should not exceed 3 days (National Health Commission, 2006). The control of esketamine nasal spray is as stringent as that of narcotic drugs. At least for now, the esketamine nasal spray has not been used on a large scale in hospitals in mainland China, although nearly 1 year has passed since the NMPA agreed to its approval.

It is worth mentioning that the indication approval of esketamine nasal spray is tortuous. The FDA’s approval of antidepressants usually requires two successful Phase III short-term trials (Turner, 2019). However, among the three short-term trials of esketamine nasal spray, two results were not better than those of the placebo group. Therefore, the FDA accepted only one successful trial and added another effect maintenance study (Horowitz and Moncrieff, 2021). Based on this, the initial indication is limited to adult with TRD. In August 2020, the FDA extended the indication of esketamine nasal spray to adults with MDD and suicidal ideation.

**FIGURE 2:**
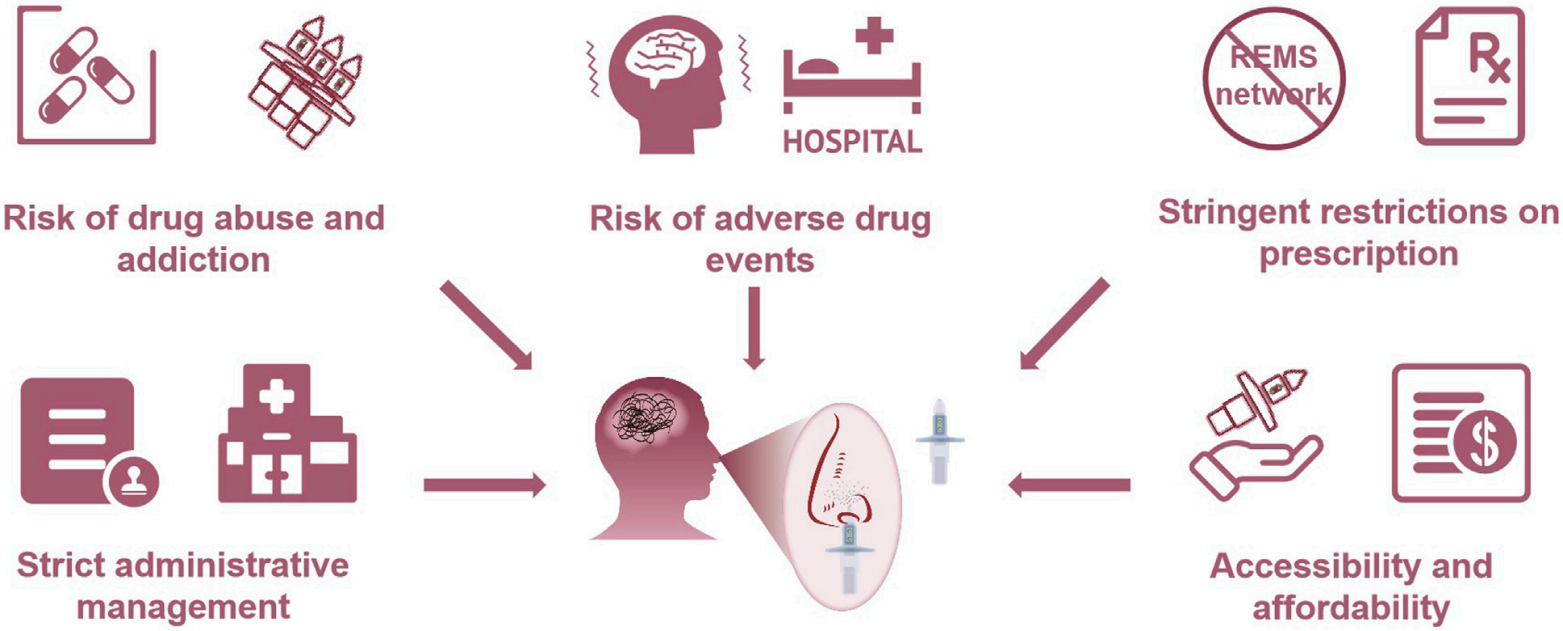
Challenges for esketamine nasal spray in China.

In China, esketamine nasal spray is used to alleviate depressive symptoms in combination with oral antidepressants in patients with depression and acute suicidal ideation or behavior. It emphasizes the quick remission of acute suicidal ideation or behavior. Notably, the NMPA emphasizes that the efficacy of esketamine nasal spray in preventing suicide or reducing suicidal ideation or behavior has not been fully confirmed. A Phase III short-term clinical trial only is insufficient to support its clinical effectiveness. Although the NMPA made concessions due to the new mechanism of this antidepressant and approved it rapidly, they do have concerns regarding its efficacy. Furthermore, even if the patient’s symptoms improved after the initial administration, the NMPA stressed the requirement for hospitalization if required clinically, implying that esketamine nasal spray is likely to be exclusively used to manage depression-related suicidal symptoms in China. In clinical practice, it is almost difficult for patients with suicidal behavior to obtain this drug if they are not in the hospital. If the patient is in hospital, there are many other measures that can reduce suicidal ideation and behavior. The dilemma of esketamine nasal spray in China is the contradiction between the feasibility of reducing depressive symptoms and the clinical administrative control of abuse prevention. Finally, the drug must be administered under the direct supervision of the healthcare provider, which increased the workload of grassroots medical staff in China (Figure 2).

## Economic considerations and accessibility

The audiences of esketamine nasal spray in China are all patients with depression who need it (Canuso et al., 2018). However, it may not be affordable. In the United States, esketamine nasal spray therapy is not a cost-effective treatment, with $700–$900 for a 56 mg dose and $1,100–$1,400 for an 84 mg dose each time, and the cost for a 1-month induction phase of esketamine therapy ranges approximately from $5,000 (56 mg dose) to $7,500 (84 mg dose) (Jalloh, 2020). As China’s largest payer of medical expenses, the National Healthcare Security Administration might not be able to bear such high costs. Furthermore, the medical security capacity is relatively low in the rural areas of China, with 75.4% of patients with depression not receiving professional treatment, and the incidence of depression is higher than that in urban areas (Li et al., 2014). It is difficult for patients living in rural areas of China to access esketamine nasal spray in REMS-certified institutions, not to mention whether they can afford the high cost. If stringent drug abuse control is performed satisfactorily, esketamine nasal spray should be actively allocated to rural primary health institutions, where the disease burden is the largest, and patients with low socioeconomic status are unlikely to seek depression treatment actively. Esketamine nasal spray should cover areas with high incidence of depression to bridge the contradiction between China’s limited medical resources and high depression groups. Besides, medical insurance, manufacturers, and third-party payment systems should actively follow up to share medical expenses through multiple channels. Otherwise, the introduction of esketamine nasal spray might aggravate regional economic disparity in depressive disorders. There is an urgent need to increase efforts to address these issues in order to achieve high accessibility of this drug and ultimately reduce suicidal behavior caused by depression in China.
